# Identification of gene expression and DNA methylation of *SERPINA5* and *TIMP1* as novel prognostic markers in lower-grade gliomas

**DOI:** 10.7717/peerj.9262

**Published:** 2020-06-03

**Authors:** Wen-Jing Zeng, Yong-Long Yang, Zhi-Peng Wen, Peng Chen, Xiao-Ping Chen, Zhi-Cheng Gong

**Affiliations:** 1 Department of Clinical Pharmacology, Xiangya Hospital, Central South University, Changsha, Hunan, China; 2 Institute of Clinical Pharmacology, Central South University, Hunan Key Laboratory of Pharmacogenetics, Changsha, Hunan, China; 3 Department of Pharmacy, Xiangya Hospital, Central South University, Changsha, Hunan, China; 4 National Clinical Research Center for Geriatric Disorders (XIANGYA), Xiangya Hospital, Central South University, Changsha, Hunan, China; 5 Department of Clinical Pharmacology Research Center, Changsha Carnation Geriatrics Hospital, Changsha, Hunan, China

**Keywords:** Lower-grade glioma, SERPINA5, TIMP1, DNA methylation, Bioinformatics analysis, Prognostic markers

## Abstract

**Background:**

Lower-grade gliomas (LGGs) is characteristic with great difference in prognosis. Due to limited prognostic biomarkers, it is urgent to identify more molecular markers to provide a more objective and accurate tumor classification system for LGGs.

**Methods:**

In the current study, we performed an integrated analysis of gene expression data and genome-wide methylation data to determine novel prognostic genes and methylation sites in LGGs.

**Results:**

To determine genes that differentially expressed between 44 short-term survivors (<2 years) and 48 long-term survivors (≥2 years), we searched LGGs TCGA RNA-seq dataset and identified 106 differentially expressed genes. *SERPINA5* and* TIMP1* were selected for further study. Kaplan–Meier plots showed that *SERPINA5* and *TIMP1* expression were significantly correlated with overall survival (OS) and relapse-free survival (RFS) in TCGA LGGs patients. We next validated the correlation between the candidate genes expression and clinical outcome in CGGA LGGs patients. Multivariate analysis showed that *TIMP1* mRNA expression had a significant prognostic value independent of other variables (HR = 4.825, 95% CI = 1.370–17.000, *P* = 0.014). Then, differential methylation sites were identified from differentially candidate gene expression groups, and all four methylation sites were significantly negatively correlated with gene expression (spearman *r* <  − 0.5, *P* < 0.0001). Moreover, hyper-methylation of four methylation sites indicated better OS (*P* < 0.05), and three of them also shown statistical significantly association with better RFS, except for *SERPINA5* cg15509705 (*P* = 0.0762).

**Conclusion:**

Taken together, these findings indicated that the gene expression and methylation of *SERPINA5* and *TIMP1* may serve as prognostic predictors in LGGs and may help to precise the current histology-based tumors classification system and to provide better stratification for future clinical trials.

## Introduction

Gliomas are the most common primary malignancies of the central nervous system that include astrocytoma, ependymoma, oligodendroglioma, and mixed oligoastrocytomas, and ranged in grade II to IV as defined by the World Health Organization (WHO). Because of its histopathological heterogeneity, the clinical outcome of glioma patients varies widely (Louis et al., 2007; Louis et al., 2016). Lower-grade gliomas (LGGs), comprising WHO grades II and III astrocytoma, oligodendroglioma and mixed oligoastrocytoma, exhibit infiltrative and highly invasive nature and intrinsic tendency to recur or progress into WHO grade IV gliomas (Brat et al., 2015). Despite recent advances in neurosurgery, radiotherapy and chemotherapy, LGGs patients have a wide range of survival, ranging from 1 to 15 years (Brat et al., 2015; Ceccarelli et al., 2016; Ricard et al., 2012). Currently, biomarkers used to treat and predict the prognosis of LGGs are limited (Brennan et al., 2013; Louis et al., 2014), so it is urgent to identify more molecular markers to provide a more objective and precise tumor classification system for LGGs.

The genetic and epigenetic landscapes of LGGs have been extensively studied (Brat et al., 2015; Chan, Mao & Ng, 2016; Suzuki et al., 2015). Transcriptomic data, one of the most commonly available high-throughput molecular data, plays a critical role in identifying novel tumor genetic biomarker and discovering new drug targets. Verhaak et al. (2010) analyzed gene expression data of glioblastoma (GBM) and classified GBM into Proneural, Neural, Classical, and Mesenchymal subtypes. Weller et al. (2015) analyzed transcriptome-wide data of primary tumor samples identified eight transcriptionally different groups (five isocitrate dehydrogenase (IDH)1/2 mutant, three IDH1/2 wild type). Recently, studies have revealed that IDH mutations disrupt histone demethylation and suggest a better survival rate (Lu et al., 2012). In particular, IDH mutation and 1p/19q deletion are used as biomarkers to classify gliomas in the 2016 WHO classification of central nervous system tumors (Louis et al., 2016). However, current molecular classification does not guarantee accurate diagnosis and individualized medication for LGG patients.

DNA methylation, an epigenetic modification via methylation of cytosin carbon 5, is an important epigenetic modification related to the pathogenesis of gliomas (Ellis, Atadja & Johnstone, 2009; Herman et al., 1996; Noushmehr et al., 2010). Previous evidences demonstrated that the increased methylation of DNA in 5*’* upstream regulatory sites negatively correlate with gene expression of some tumor-suppression genes (Merlo et al., 1995). It is widely recognized that the activity of DNA-repair enzyme O (sup 6)- methylguanine-DNA methyltransferase (MGMT) is controlled by its promoter methylation status, which can effectively predict the responsiveness of the gliomas to alkylating agents (Esteller et al., 2000; Hegi et al., 2005). These evidences suggested that alteration of DNA methylation can be exploited for functional characterizations and diagnosis of gliomas. However, there is still no clear understanding of the epigenetic alterations in LGGs, and of the potential role of DNA methylation markers as prognostic biomarkers.

In the present study, we performed an integrated analysis of gene expression data and DNA methylation data from The Cancer Genome Atlas (TCGA) and Chinese Glioma Genome Atlas (CGGA) databases to determine novel prognostic genes and methylation sites in LGGs. We found that the gene expression and methylation of *SERPINA5* and *TIMP1* can function as prognostic predictors in LGGs, which might help to precise the current histology-based tumors classification system and to provide better stratification for future clinical trials.

## Materials and Methods

### Lower-grade glioma datasets

TCGA LGG dataset was downloaded from the University of California Santa Cruz cancer browser https://genome-cancer.ucsc.edu/ (version: 2015-02-24) as training dataset. In total, 473 samples (225 grade II, 248 grade III gliomas) having clinical data were profiled for class discovery and survival analysis. A total of 131 samples (97 grade II, 34 grade III gliomas) from CGGA repository (http://cgga.org.cn/) was included in our analysis as validation dataset, and all samples*’* clinical data were downloaded for survival analysis. Overall survival (OS) was defined as the time interval from resection until the date of death. Relapse-free survival (RFS) is the period from resection to the radiological evidence of first tumor recurrence.

### Gene expression data analysis

Gene expression data of TCGA LGG are from the Illumina HiSeq 2000 RNA Sequencing platform, and all counts data is then log2(count+1) transformed. The differential gene expression analysis and the adjusting for multiple testing was performed with edgeR package (Robinson, McCarthy & Smyth, 2010). The CGGA microarray dataset was generated by Agilent Whole Human Genome Array, and probe intensities were normalized using GeneSpring GX 11.0 (Yan et al., 2012). In the TCGA LGG dataset, 299 patients were classified as short-term survivors (<2 years) and the remaining 174 patients as long-term survivors (≥2 years). In order to exclude the influence of loss of follow-up or short follow-up time, we excluded the samples that did not reach the end time, and only samples that had died and had a clear survival time were included for screening of differential expressed genes (DEGs). Thus, a total of 44 short-term survivors (<2 years) and 48 long-term survivors (≥2 years) were included in the gene differential expression analysis. Genes were considered to have significant difference in expression if —log fold change (FC)— ≥ 1.0 and adjusted *P* < 0.05. The prognostic value of DEGs generated from TCGA lower-grade glioma dataset were then validated using Kaplan–Meier survival analysis with the CGGA LGG microarray dataset.

### DNA methylation analysis

DNA methylation data of TCGA LGG are generated by the Illumina Infinium HumanMethylation450 platform. Mapping between probes on the RNA-seq and DNA methylation probes on the methylation array was performed. The β value was used to estimate the methylation level of probes. Probes with β ≥0.5 was considered as hyper-methylated sites, and β <0.5 was considered as hypo-methylated sites. The correlation between gene expression levels and DNA methylation levels were assessed using Spearman’s correlation analysis. —Spearman r— ≥0.6 was indicated a strong correlation and *P* < 0.05 was considered as statistically significant (Zeng et al., 2018).

To investigate the correlation between gene expression and DNA methylation, we performed a parallel DNA methylation analysis of the candidate genes. Mapping *SERPINA5* and *TIMP1* to DNA methylation probes identified 23 and 14 methylation sites, respectively. To obtain differentially methylated sites, patients were divided into two groups according to the median of gene expression. Of the 37 methylation sites, 4 differential methylation sites (*SERPINA5* cg15509705; *TIMP1* cg27151711; *TIMP1* cg16523424; *TIMP1* cg04791822) were identified (Table S2).

### Functional enrichment analysis

The Database for Annotation, Visualization and Integrated Discovery (DAVID) was used to identify the potential biological functions of co-differentially expressed genes (co-DEGs) in LGGs (https://david.ncifcrf.gov/home.jsp) (Huang da, Sherman & Lempicki, 2009; Yan et al., 2019). Gene ontology (GO) analysis, including biological processes (BP), molecular function (MF), and cellular composition (CC), was performed to annotate these genes and determine functional enrichment. *P* <0.05 was considered as statistically significant for pathway enrichment. Then, the protein and protein interaction network of SERPINA5 and TIMP1 were constructed with GENEMANIA online database (https://genemania.org/) (Warde-Farley et al., 2010; Zhang et al., 2017).

### Statistical analysis

Statistical analyses were performed with SPSS 13.0 (SPSS Inc., Chicago, IL, USA) and GraphPad Prism 5.0 (Graphpad Inc., San Diego, CA, USA). Nonparametric test was performed to identify genes that were differentially expressed between two groups. Spearman’s correlation analysis was used to determine the correlation between gene expression levels and DNA methylation status. Kaplan–Meier survival analysis was carried out to assess the survival distribution and the log-rank test was performed to determine the significance of the differences between two groups (Wang et al., 2019). For multivariable analysis, a Cox proportional hazards model was constructed for OS with a limited backward-LR procedure and was adjusted by potential confounding covariates. Hazard ratio (HRs) and 95% confidence intervals (CIs) were used to describe the risk. *P* <0.05 was considered as statistically significant.

## Results

### Identification of prognostic DEGs in LGGs (Fig. 1)

**Figure 1:**
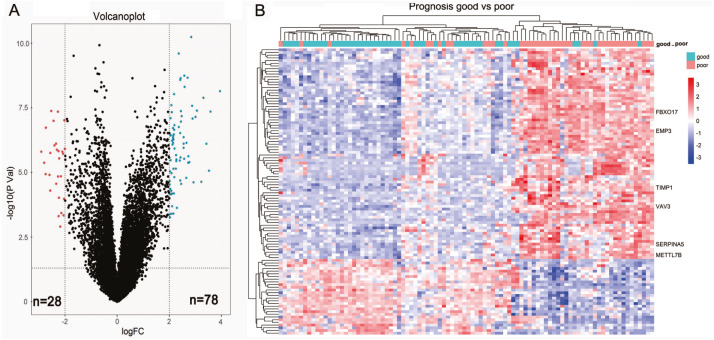
Identification of differential expressed genes between short-term survivors (<2 years) and long-term survivors (≥2 years) in the lower-grade gliomas in the TCGA microarray dataset. (A) Volcano plot of the differential genes expression analysis. (B) Hierarchical cluster analysis of the mRNA expression profiles of short-term survivors and long-term survivors.

We first sought to identify DEGs between 44 short-term survivors (<2 years) and 48 long-term survivors (≥2 years) in the LGGs of TCGA microarray dataset. In total, 106 genes (78 upregulated genes and 28 down-regulated genes) were identified to be differentially expressed (Fig. 1A, Table 1). The results of two-dimensional hierarchical cluster indicated that the mRNA expression profiles of short-term survivors and long-term survivors distributed in separate clusters (Fig. 1B).

**Table 1:** Differential expressed genes between short-term survivors (<2 years) and long-term survivors ( ≥2 years) in the lower-grade gliomas in the TCGA microarray dataset.

	**Differentially expressed genes (DEGs)**
Up-regulated genes	EMP3, FBXO17, **SERPINA5**, VAV3, IGFBP2, **TIMP1**, METTL7B, CHI3L1, GALNT3, ABCC3, ZDHHC23, STAC, AQP5, CMYA5, MOXD1, FBLN7, HILS1, HOXA3, VASN, HOXA5, TOM1L1, MAP1LC3C, C13orf26, EVC2, WISP1, RARRES2, PDLIM4, SHROOM3, NPNT, RBP1, NKX2-5, GDF15, ANXA1, IGF2BP3, ADAM12, TSTD1, FABP5, TRPM8, DSG2, MEOX2, MAOB, PLA2G5, C11orf63, RYR3, HOXB3, HOXA2, PLA2G2A, CLEC5A, LOXL1, RGS22, FMOD, SHOX2, DMRTA2, PDPN, CA3, POSTN, SAA1, WNT16, CNGA3, LGR6, HOXA4, GPR1, LTF, HOXA7, TCTEX1D1, C21orf62, HMGA2, CXCL14, OTP, EYA4, HOXD11, SLC47A2, DDIT4L, COL22A1, IL13RA2, DES, ALDH1A3, C2orf39
Down-regulated genes	ADAMTS20, CRTAC1, TMEM100, DAPL1, HMX1, WNT7B, NEUROD4, GFRA1, NDST4, FERMT1, PRLHR, DLL3, C5orf38, LPPR3, SMOC1, SFRP2, LOC154822, KLRC2, CSMD3, CUX2, PAX1, IRX2, HPSE2, SPHKAP, TSHR, psiTPTE22, TLX1, IRX1

### Functional enrichment analysis of DEGs in LGGs

To explore the potential biological functions of prognostic related DEGs in LGGs, GO categories and KEGG Pathway enrichment analysis were performed using the DAVID online database. GO analysis revealed that 106 DEGs were significantly enriched in cell components such as proteinaceous extracellular matrix, extracellular space, extracellular exosome and basement membrane, and was involved in the biological processes such as multicellular organism development, thyroid gland development and anterior/posterior pattern specification (Table S1 ). KEGG Pathway enrichment analysis showed that DEGs mainly enriched in phenylalanine metabolism and histidine metabolism, but the result was not significant (Table S1).

### Survival value of top significant DEGs in LGGs

**Figure 2:**
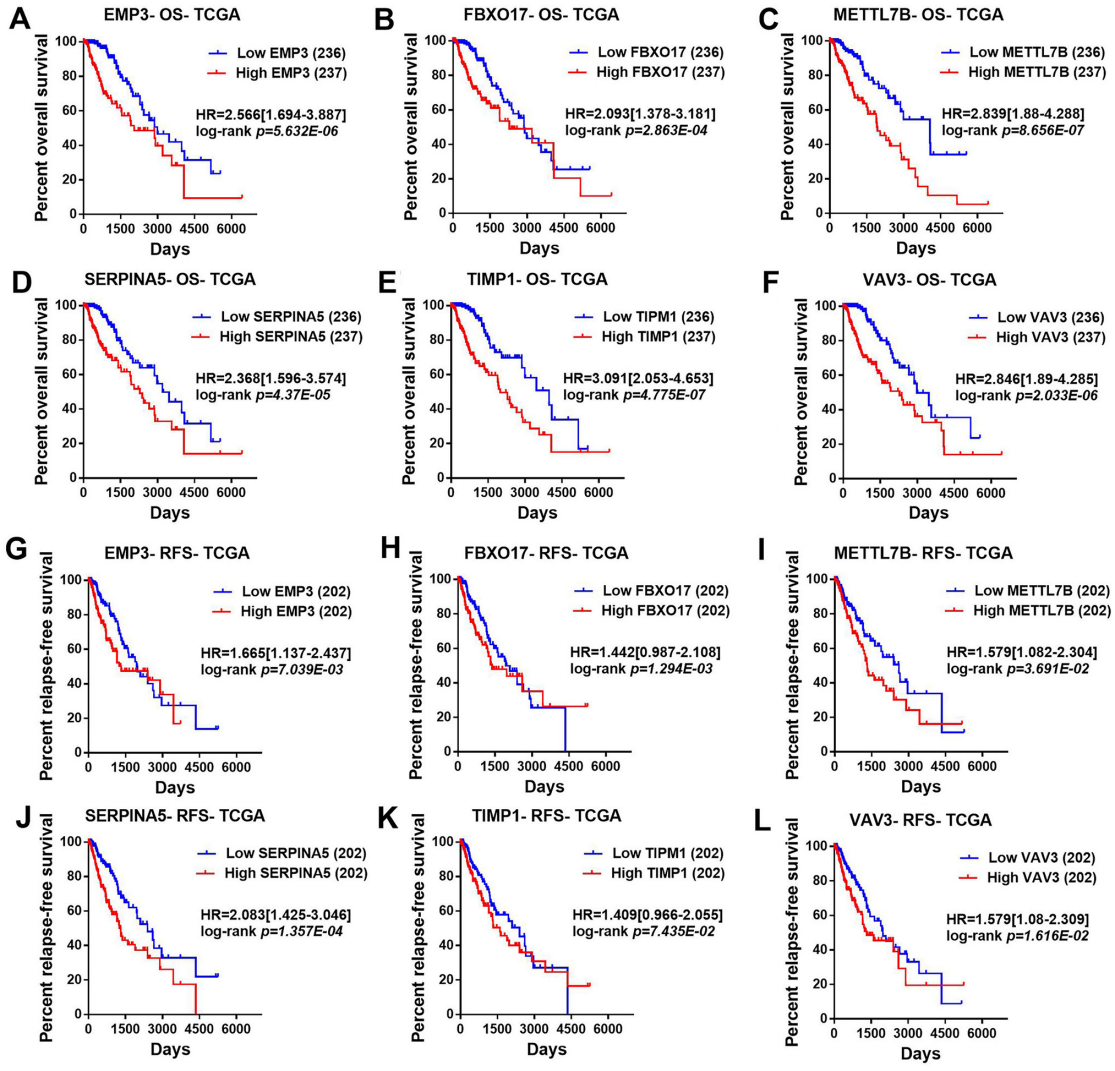
Correlation between the top six significant changed genes’ expression and patients’ survival in lower-grade glioma with TCGA LGG dataset. (A–F) Kaplan–Meier plot for overall survival between LGG patients with high level and low level of *EMP3*, *FBXO17*, *METTL7B*, *SERPINA5*, *TIMP1*, *VAV3* mRNA expression in TCGA LGG dataset. (G–L) Kaplan–Meier plot for relapse-free survival between LGG patients with high level and low level of *EMP3*, *FBXO17*, *METTL7B*, *SERPINA5*, *TIMP1*, *VAV3* mRNA expression in TCGA LGG dataset.

**Figure 3:**
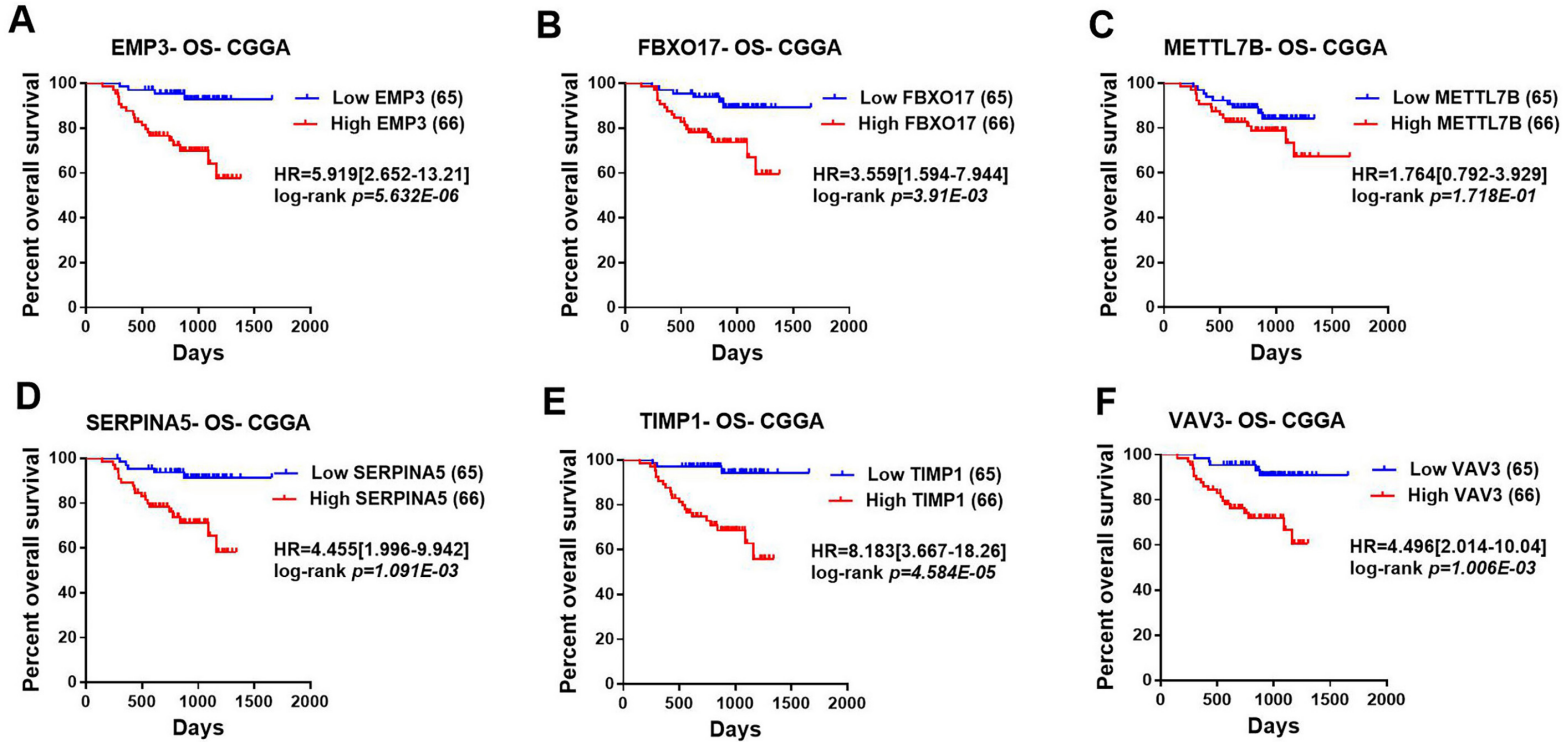
Correlation between the top six significant changed genes’ expression and patients’ survival in lower-grade glioma with CGGA dataset. (A–F) Kaplan–Meier plot for overall survival between patients with high level and low level of *EMP3*, *FBXO17*, *METTL7B*, *SERPINA5*, *TIMP1*, *VAV3* mRNA expression in CGGA dataset.

**Figure 4:**
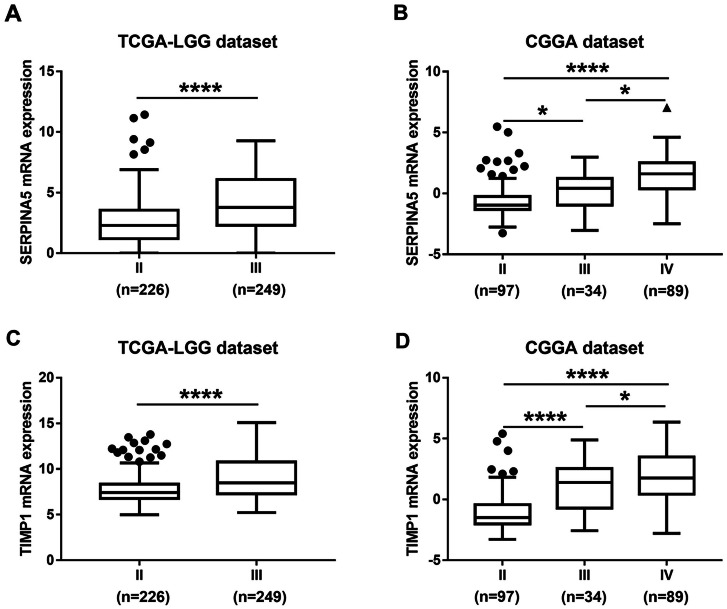
*SERPINA5* and *TIMP1* mRNA expression in glioma tissues. ****(A, B) The mRNA expression of *SERPINA5* in grade II or grade III patients in TCGA LGG and CGGA LGGs cohort. The results are mean ± SD, ^∗^*P* < 0.05, ^∗∗∗∗^*P* < 0.0001. (C, D) The mRNA expression of *TIMP1* in grade II or grade III patients in TCGA LGG and CGGA LGGs cohort. The results are mean ± SD, ^∗^*P* < 0.05, ^∗∗∗∗^*P* < 0.0001.

Then, TCGA-LGG and CGGA-LGG datasets were used to verify the prognostic value of the top eight significant changed genes, including *EMP3*, *FBXO17*, *METTL7B*, *SERPINA5*, *SSTR5*, *TIMP1*, *TMEM61*, *VAV3*. In the TCGA LGG dataset, *SERPINA5* high expression indicated the worse OS and RFS of LGG patients (Figs. 2D, 2L). However, *TIMP1* high expression can only predict the shorter OS of patients and has no significant correlation with the RFS of LGG patients (Figs. 2F, 2N). Moreover, the expression of *SERPINA5* and *TIMP1* in the CGGA dataset were coincident with those of TCGA dataset, with significant differences in OS (Fig. 3). Additionally, *SERPINA5* and *TIMP1* mRNA expression were significantly increased with the increase of glioma grades in both TCGA LGG dataset and CGGA dataset (Fig. 4).

The association between expression of the candidate genes and clinical characteristics in CGGA lower-grade glioma patients is presented in Table 2. Univariate analysis indicated that karnofsky performance score (KPS) ≥70 was significantly associated with better survival in patients (HR = 0.127, 95% CI [0.037–0.434], *P* = 0.001), and tumor grade (grade III vs grade II) was significantly correlated with poor survival in patients (HR = 8.883, 95% CI [3.670–21.501], *P* = 0.000001). Multivariate analysis, adjusted for KPS and tumor grade, also showed that the top two DEGs were significantly associated with survival. *TIMP1* high expression group exhibited poor survival as compared to low expression group (HR = 8.656, 95% CI [2.578–29.060], *P* = 0.014). Unfortunately, *SERPINA5* high expression was not an independent poor predictor for OS of LGGs patients (HR = 0.473, 95% CI [0.192–1.164], *P* = 0.103).

### DNA promoter hypermethylation silences *SERPINA5* and *TIMP1* mRNA expression

**Figure 5:**
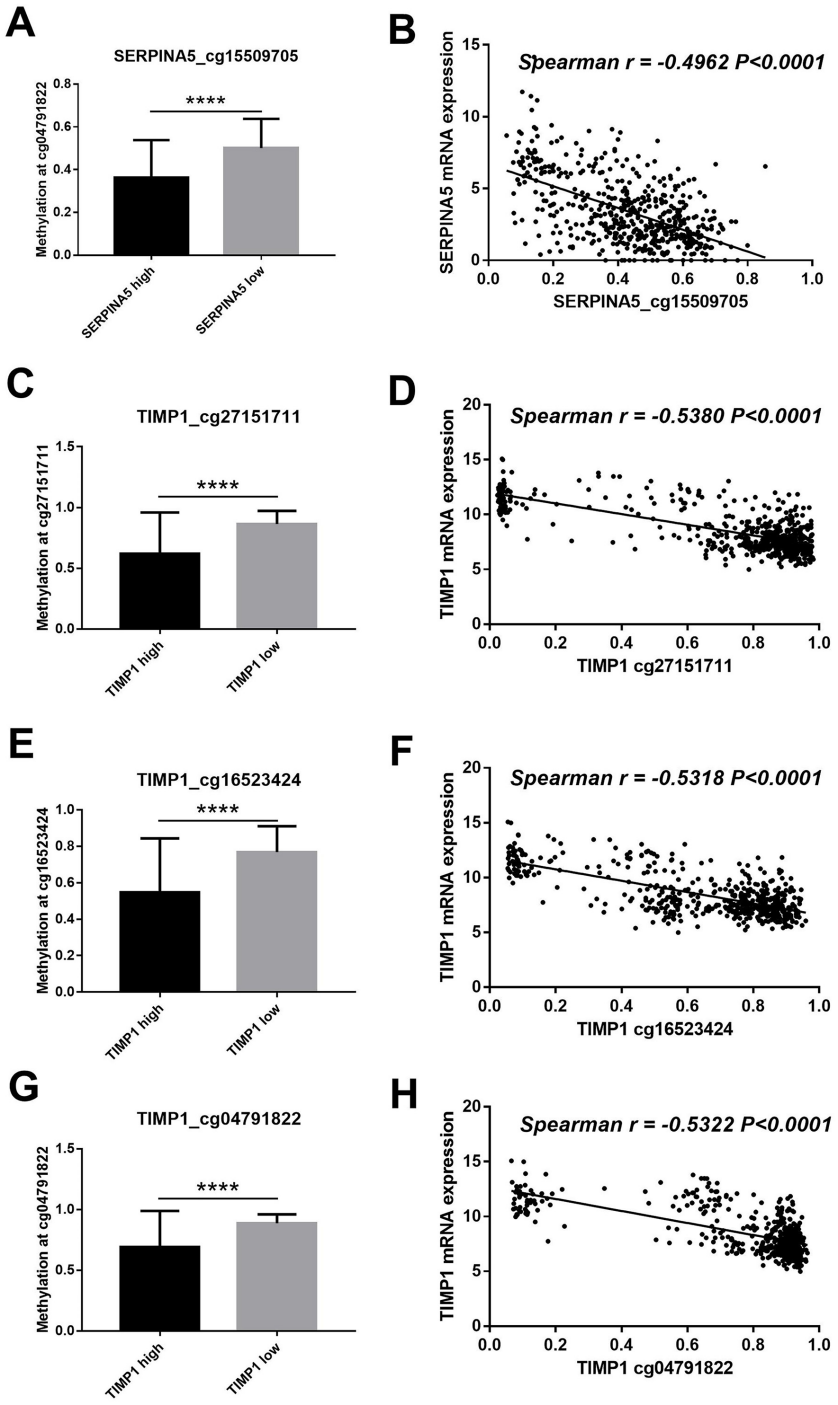
DNA methylation of *SERPINA5* and *TIMP1* is related to gene expression. (A, C, E, G) According to the median of gene expression, LGGs samples were divided into two groups to obtain differential methylation site between two groups. The results are mean ± SD, ^∗∗∗∗^*P* < 0.0001. (B, D, F, H) The correlation between*****SERPINA5* and *TIMP1* gene expression levels and DNA methylation levels of CpG sites were assessed using Spearman’s correlation analysis.

To investigate the correlation between gene expression and DNA methylation, we performed a parallel DNA methylation analysis of the candidate genes. Mapping *SERPINA5* and *TIMP1* to DNA methylation probes identified 23 and 14 methylation sites, respectively. To obtain differentially methylated sites, patients were divided into two groups according to the median of gene expression. Of the 37 methylation sites, 4 differential methylation sites (*SERPINA5* cg15509705; *TIMP1* cg27151711; *TIMP1* cg16523424; *TIMP1* cg04791822) were identified (Table S2). As shown in Fig. 5, the methylation status of 4 methylation sites were remarkably lower in high gene expression group than low gene expression group. Furthermore, the correlation analysis revealed that 4 methylation sites were negatively correlated with gene expression levels (Spearman *r* <  − 0.4, *P* < 0.0001, Fig. 5).

### *SERPINA5* and *TIMP1* methylation are potent prognostic markers for LGGs

**Figure 6:**
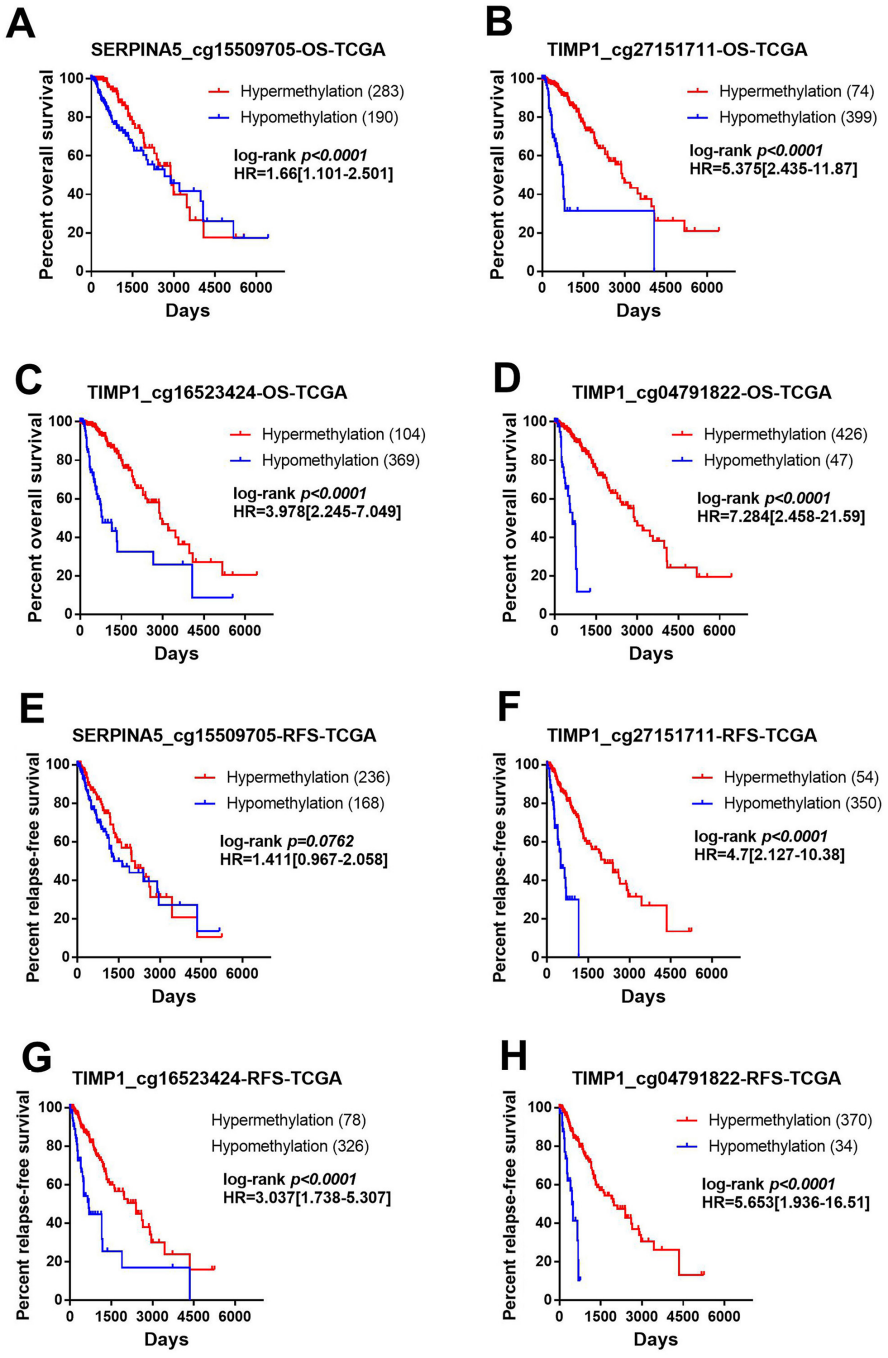
Methylation of *SERPINA5* and *TIMP1* CpG sites is associated with the survival of LGGs patients. Samples were divided into two groups with methylation beta value of 0.5 as the cut-off value and compared the difference of prognosis between the two groups. (A–D) Kaplan–Meier survival curves for overall survival between differentially methylated status of *SERPINA5* and *TIMP1* and patients in the TCGA LGG patients. (E–H) Kaplan–Meier plot for relapse-free survival between patients with hyper-methylation and hypo-methylation of *SERPINA5* and *TIMP1* in TCGA LGG patients.

In order to identify the effect of these methylation sites on prognosis, we assessed the association between 4 methylation sites and prognosis with the TCGA LGG DNA methylation dataset. The samples were divided into two groups with methylation β value of 0.5 as the cut-off value and the prognostic difference were compared. As shown in Figs. 6A–6D, hyper-methylation of 4 methylation sites indicated better OS (*SERPINA5* cg15509705: HR = 1.66, 95% CI [1.101–2.501], *P* < 0.0001; *TIMP1* cg27151711: HR = 5.375, 95% CI [2.435–11.87], *P* < 0.0001; *TIMP1* cg16523424: HR = 3.978, 95% CI [2.245–7.049], *P* <0.0001; *TIMP1* cg04791822: HR = 7.284, 95% CI [2.458–21.59], *P* <0.0001). In addition, except for *SERPINA5* cg15509705 (HR = 1.411, 95% CI [0.967–2.058], *P* = 0.0762, Fig. 6E), hypo-methylation of 3 other methylation sites the high-risk group exhibited significantly worse RFS (*TIMP1* cg27151711: HR = 4.700, 95% CI [2.127–10.38], *P* <0.0001; *TIMP1* cg16523424: HR = 3.037, 95% CI [1.738–5.307], *P* < 0.0001; *TIMP1* cg04791822: HR = 5.653, 95% CI [1.936–16.51], *P* < 0.0001, Figs. 6F–6H).

### Functional enrichment analysis of SERPINA5- and TIMP1-associated co-expressed genes

**Figure 7:**
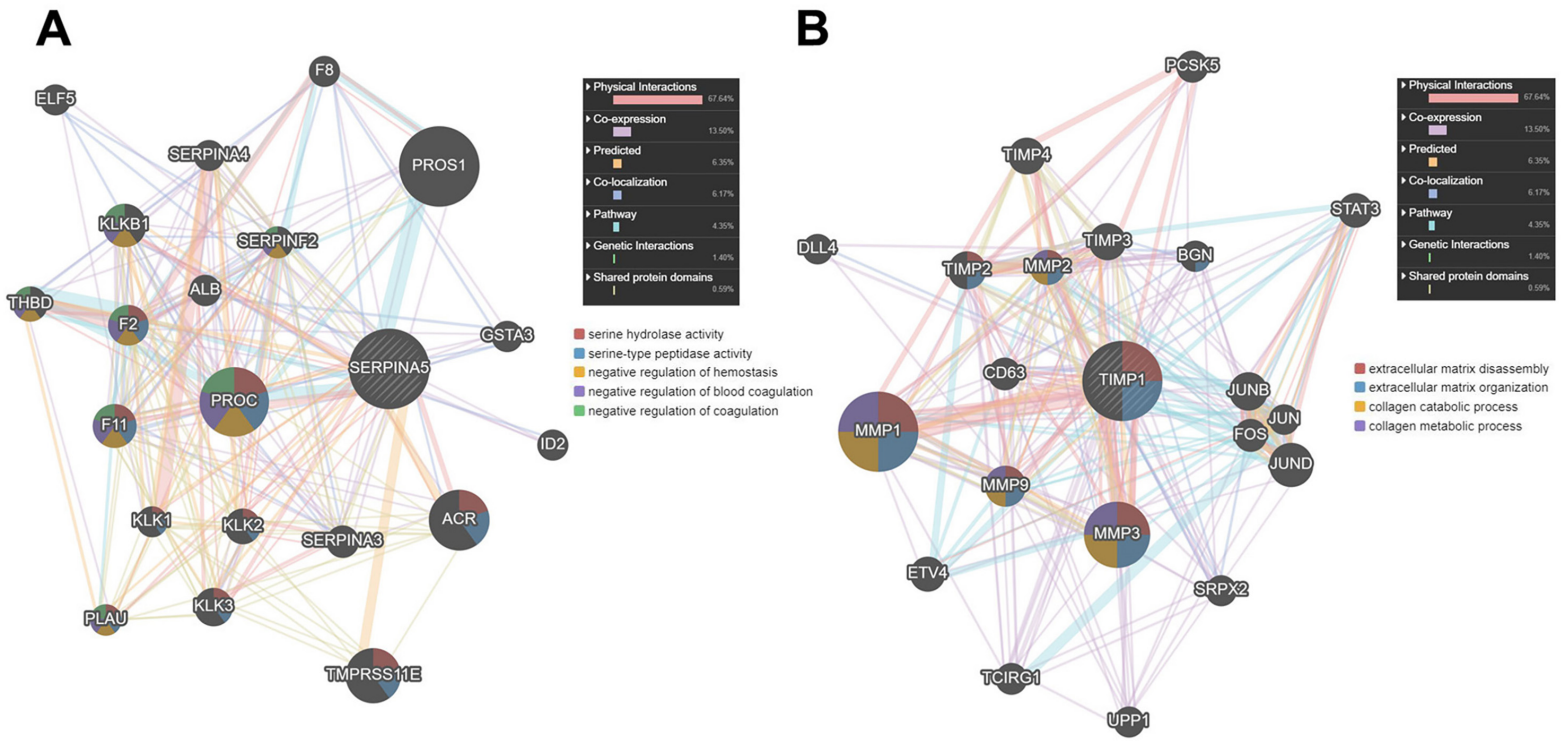
The PPI network of SERPINA5 and TIMP1 generated by the GENEMANIA online database.

Then, we used GENEMANIA online database to identify the proteins interacting with SERPINA5 and TIMP1. As shown in Fig. 7A, SERPINA5 mainly interacts with PROC, which has serine hydrolase activity and functions as a negative regulation of hemostasis and coagulation. While TIMP1 interacts with extracellular matrix proteins MMP1, MMP3 and MMP9, and participants in the processes of extracellular matrix disassembly and organization, collagen catabolism and metabolism (Fig. 7B).

**Table 2:** Associations of SERPINA5 and TIMP1 expression with clinical variables in CGGA dataset.

**Overall survival**	**Univariate**		**Multivariate**	
	***P*-value**	**HR [95% CI]**	***P*-value**	**HR [95% CI]**
Age	0.066	1.037[0.998–1.078]	ns	–
Gender: male vs female	0.474	1.342[0.599–3.006]	ns	–
Histology, Astrocytoma	0.104	2.024[0.866–4.731]	ns	–
Oligoastrocytoma/Oligodendroglioma			ns	–
KPS: ≥70 vs <70	**0.001**	0.127[0.037–0.434]	ns	–
IDH1: mut vs wild type	0.305	0.656[0.293–1.467]	ns	–
Tumor grade: Grade ?vs Grade?	**0.000001**	8.883[3.670–21.501]	**0.000257**	5.551[2.214–13.915]
SERPINA5 expression: high vs low	**0.103**	0.473[0.192–1.164]	ns	–
TIMP1 expression: high vs low	**0.000478**	8.656[2.578–29.060]	**0.014**	4.825[1.370–17.000]

**Notes.**

Bold font indicates statistical significance.

KPS karnofsky performance score IDH1 isocitrate dehydrogenase 1 SERPINA5 Serpin family A member 5 TIMP1 TIMP Metallopeptidase Inhibitor 1 HR Hazard ratio CI confidence intervals

## Discussion

This study was conducted to identify DEGs between long-term and short-term survivors in LGGs with TCGA LGG RNA-seq dataset and we obtained 106 DEGs, among which *SERPINA5* and *TIMP1* were differentially expressed. Since removing the “the samples that did not reach the end time” might skew the results, we analyzed the differentially expressed genes without removing any data, and *SERPINA5* and *TIMP1* were also differentially expressed between short-term and long-term survivors in LGGs (*SERPINA5*: log FC = 0.833, adjusted *P* = 0.00488; *TIMP1*: log FC = 0.788, adjusted *P* = 0.00253). Thus, this study focused on these two genes.

*SERPINA5* (protein C inhibitor, PCI) is a member of serine protease inhibitor super family, which can inhibit several serine proteases, including protein C and various plasminogen activators and kallikreins, and it thus plays diverse roles in hemostasis and thrombosis in multiple organs (Yang & Geiger, 2017). Extracellular matrix degradation is facilitated by uPA, allowing tumor cells to invade surrounding tissue (Fortenberry, 2015). SerpinA5 is an uPA inhibitor that prevents the conversion of plasminogen to plasminogen and subsequent extracellular matrix degradation (Smith & Marshall, 2010). Previous studies indicated that the dysregulation of *SERPINA5* has been implicated in migration, invasion and metastasis in hepatocellular carcinoma, ovarian and prostate cancers (Bijsmans et al., 2011; Cao et al., 2003; Jing et al., 2014). However, the roles of *SERPINA5* in gliomas remains unknown. In this study, we found that *SERPINA5* expression was significantly correlated with OS and RFS in LGG patients, and *SERPINA5* high expression indicated patients with worse survival. More recently, researchers have found that the high methylation degree of CpG sites significantly correlated with lower *SERPINA5* expression levels and two distinct CpG sites of the *SERPINA5* promoter were hypermethylated in normal epithelial prostate cells, benign hyperplasic cells and low-invasive malignant LNCaP cells, whereas essentially unmethylated in aggressive DU-145 and PC-3 cell line (Hagelgans et al., 2017). In addition, *SERPINA5* has been identified to be more highly methylated in HR-, basal-like, or p53 mutant breast cancer than HR+, luminal A, or p53 wild-type breast cancers, and gene signature composed of SERPINA5 and 3 other genes can predict the prognosis of patients with stage I LUAD (Conway et al., 2014; Luo, Wang & Zhang, 2018). These evidences suggested that methylation of *SERPINA5* may be used to indicate the malignancy of some tumors and to predict the prognosis of patients. In this study, our results also shown that hyper-methylation of *SERPINA5* was statistical significantly association with lower expression and better prognosis in LGGs. Previous research has reported that the expression of coagulation inhibitors PRCO and SERPINA5 is strongly regulated by sex-specific GH patterns (Wong et al., 2008). While the exact role of SERPINA5 in glioma progression remains to be determined.

Experimental studies have demonstrated the contribution of TIMPs to the majority of cancer hallmarks, and human cancers invariably have shown TIMP deregulation in the tumor or stroma (Jackson et al., 2017). Previous studies reported that the characteristic of human neural stem cells (hNSCs) migration towards intracranial glioma is regulated by the TIMP1 (Lee et al., 2014). Moreover, researchers have shown that both serum TIMP1 level and *TIMP1* mRNA expression of glioma tissue in GBM patients were significantly higher than grade II/III patients (Sreekanthreddy et al., 2010; Xu et al., 2019), and serum angiogenic profile in GBM patients identified that the serum TIMP-1 level as an independent predictor of survival (Crocker et al., 2011). The overall relationship of high *TIMP1* expression with poor cancer outcome has been demonstrated in gliomas (Aaberg-Jessen et al., 2009). Consistent with previous studies, our results shown that *TIMP1* high expression was also independent poor predictor for OS. Additionally, we also found that methylation of *TIMP1* were highly negatively correlated with its gene expression and hyper-methylation of *TIMP1* indicated better OS and RFS. Collectively, these results suggested that *TIMP1* may be an important biomarker in glioma patient fluids and target for designing therapy. Thus, further studies should be performed to establish the exact mechanisms of TIMP1 in the tumor microenvironment and its pro-tumorigenic function in gliomas. TIMP1 mainly participants in the processes of extracellular matrix disassembly and organization (Soini et al., 2001). Further experiments are needed to explore the precise role of TIMP1 in glioma progression, and the potential application for the novel treatment of LGGs.

Previous evidences indicated that dysregulation of F-box protein-mediated ubiquitylation has been implicated in cancer and other diseases (Duan et al., 2012; Frescas & Pagano, 2008). In this study, we also found that *FBXO17* high expression indicated LGGs patients with worse survival. Moreover, our results shown that hyper-methylation of *FBXO17* was statistical significantly association with lower expression and better prognosis in LGGs (Fig. S1 ). In addition, it has been reported that EMP3 high expression is associated with a worse prognostic significance in OS in glioma patients (Gao et al., 2016; Zeng et al., 2018). Consistent with previous studies, our study also supported an oncogenic role on the part of EMP3 in glioma.

*IDH1/2* mutations are clearly important prognostic markers in gliomas (Sanson et al., 2009; Weller et al., 2009; Yan et al., 2009). In LGGs, patients with *IDH1/2* mutation have better prognosis than patients with *IDH* wild-type (Lu et al., 2012). In anaplastic oligodendroglial tumors, *IDH1* mutation are prognostic for overall survival but not predictive for outcome to PCV chemotherapy (Van den Bent et al., 2010). In this study, we analyzed the prognostic value of *IDH* mutation for LGG patients using CGGA LGG dataset and found that there was no significant difference in overall survival between *IDH* mutation and *IDH* wild-type patients, which may be caused by the unbalanced distribution of 1p/19q co-deletion, MGMT methylation and the TCGA molecular subtypes. Therefore, it is a limitation that we did not compare the prognostic value of the proposed markers (SERPINA5 and TIMP1) with the previously recommended or currently used marker IDH1.

## Conclusions

In this study, we identified *SERPINA5* and *TIMP1* as prognostic predict markers in LGGs, and the methylation of these genes is correlated with the survival of LGG patients. Our research indicated that both genes expression strongly correlated with methylation level are more likely to be associated with cancer outcomes. In addition, the present study firstly revealed that *SERPINA5* hypo-methylation was negatively correlated with its expression, and both hypo-methylation and high expression of *SERPINA5* predict poor survival in LGGs.

##  Supplemental Information

10.7717/peerj.9262/supp-1 Figure S1 Methylation of *FBXO17* CpG sites is associated with its expression and survival of LGGs patients (A–C) Kaplan–Meier plot for survival between patients with high level and low level of *FBXO17* expression in TCGA LGG and CGGA LGG dataset. (D) DNA methylation of *FBXO17* CpG sites correlates with its gene expression in TCGA LGG dataset. (E, F) Methylation of *FBXO17* CpG sites is associated with survival of LGGs patients in TCGA LGG dataset

10.7717/peerj.9262/supp-2 Table S1 Gene ontology analysis of DEGs in LGGs

10.7717/peerj.9262/supp-3 Table S2 Characteristics of the selected DNA methylation probes

10.7717/peerj.9262/supp-4 Table S3 Differential expressed genes between short-term survivors (<2 years) and long-term survivors (≥2 years) in the 92 lower-grade gliomas in the TCGA microarray dataset

10.7717/peerj.9262/supp-5 Table S4 Differential expressed genes between short-term survivors (<2 years) and long-term survivors (≥2 years) in the 473 lower-grade gliomas in the TCGA microarray dataset

10.7717/peerj.9262/supp-6 Table S5 The raw data of COX regression analysis of CGGA LGG dataset

10.7717/peerj.9262/supp-7 Table S6 The raw data of correlation between DNA methylation and mRNA expression

10.7717/peerj.9262/supp-8 Table S7 Figure raw data

## Additional Information and Declarations

### Data Availability

The following information was supplied regarding data availability: The raw data is available in the Supplementary Files.

## References

[ref-1] Aaberg-Jessen C, Christensen K, Offenberg H, Bartels A, Dreehsen T, Hansen S, Schroder HD, Brunner N, Kristensen BW (2009). Low expression of tissue inhibitor of metalloproteinases-1 (TIMP-1) in glioblastoma predicts longer patient survival. Journal of Neuro- Oncology.

[ref-2] Bijsmans IT, Smits KM, De Graeff P, Wisman GB, Van der Zee AG, Slangen BF, De Bruine AP, Van Engeland M, Sieben NL, Van de Vijver KK (2011). Loss of SerpinA5 protein expression is associated with advanced-stage serous ovarian tumors. Modern Pathology.

[ref-3] Brat DJ, Verhaak RG, Aldape KD, Yung WK, Salama SR, Cooper LA, Rheinbay E, Miller CR, Vitucci M, Morozova O, Robertson AG, Noushmehr H, Laird PW, Cherniack AD, Akbani R, Huse JT, Ciriello G, Poisson LM, Barnholtz-Sloan JS, Berger MS, Brennan C, Colen RR, Colman H, Flanders AE, Giannini C, Grifford M, Iavarone A, Jain R, Joseph I, Kim J, Kasaian K, Mikkelsen T, Murray BA, O’Neill BP, Pachter L, Parsons DW, Sougnez C, Sulman EP, Vandenberg SR, Van Meir EG, Von Deimling A, Zhang H, Crain D, Lau K, Mallery D, Morris S, Paulauskis J, Penny R, Shelton T, Sherman M, Yena P, Black A, Bowen J, Dicostanzo K, Gastier-Foster J, Leraas KM, Lichtenberg TM, Pierson CR, Ramirez NC, Taylor C, Weaver S, Wise L, Zmuda E, Davidsen T, Demchok JA, Eley G, Ferguson ML, Hutter CM, Mills Shaw KR, Ozenberger BA, Sheth M, Sofia HJ, Tarnuzzer R, Wang Z, Yang L, Zenklusen JC, Ayala B, Baboud J, Chudamani S, Jensen MA, Liu J, Pihl T, Raman R, Wan Y, Wu Y, Ally A, Auman JT, Balasundaram M, Balu S, Baylin SB, Beroukhim R, Bootwalla MS, Bowlby R, Bristow CA, Brooks D, Butterfield Y, Carlsen R, Carter S, Chin L, Chu A, Chuah E, Cibulskis K, Clarke A, Coetzee SG, Dhalla N, Fennell T, Fisher S, Gabriel S, Getz G, Gibbs R, Guin R, Hadjipanayis A, Hayes DN, Hinoue T, Hoadley K, Holt RA, Hoyle AP, Jefferys SR, Jones S, Jones CD, Kucherlapati R, Lai PH, Lander E, Lee S, Lichtenstein L, Ma Y, Maglinte DT, Mahadeshwar HS, Marra MA, Mayo M, Meng S, Meyerson ML, Mieczkowski PA, Moore RA, Mose LE, Mungall AJ, Pantazi A, Parfenov M, Park PJ, Parker JS, Perou CM, Protopopov A, Ren X, Roach J, Sabedot TS, Schein J, Schumacher SE, Seidman JG, Seth S, Shen H, Simons JV, Sipahimalani P, Soloway MG, Song X, Sun H, Tabak B, Tam A, Tan D, Tang J, Thiessen N, Triche Jr T, Van Den Berg DJ, Veluvolu U, Waring S, Weisenberger DJ, Wilkerson MD, Wong T, Wu J, Xi L, Xu AW, Yang L, Zack TI, Zhang J, Aksoy BA, Arachchi H, Benz C, Bernard B, Carlin D, Cho J, DiCara D, Frazer S, Fuller GN, Gao J, Gehlenborg N, Haussler D, Heiman DI, Iype L, Jacobsen A, Ju Z, Katzman S, Kim H, Knijnenburg T, Kreisberg RB, Lawrence MS, Lee W, Leinonen K, Lin P, Ling S, Liu W, Liu Y, Liu Y, Lu Y, Mills G, Ng S, Noble MS, Paull E, Rao A, Reynolds S, Saksena G, Sanborn Z, Sander C, Schultz N, Senbabaoglu Y, Shen R, Shmulevich I, Sinha R, Stuart J, Sumer SO, Sun Y, Tasman N, Taylor BS, Voet D, Weinhold N, Weinstein JN, Yang D, Yoshihara K, Zheng S, Zhang W, Zou L, Abel T, Sadeghi S, Cohen ML, Eschbacher J, Hattab EM, Raghunathan A, Schniederjan MJ, Aziz D, Barnett G, Barrett W, Bigner DD, Boice L, Brewer C, Calatozzolo C, Campos B, Carlotti Jr CG, Chan TA, Cuppini L, Curley E, Cuzzubbo S, Devine K, DiMeco F, Duell R, Elder JB, Fehrenbach A, Finocchiaro G, Friedman W, Fulop J, Gardner J, Hermes B, Herold-Mende C, Jungk C, Kendler A, Lehman NL, Lipp E, Liu O, Mandt R, McGraw M, McLendon R, McPherson C, Neder L, Nguyen P, Noss A, Nunziata R, Ostrom QT, Palmer C, Perin A, Pollo B, Potapov A, Potapova O, Rathmell WK, Rotin D, Scarpace L, Schilero C, Senecal K, Shimmel K, Shurkhay V, Sifri S, Singh R, Sloan AE, Smolenski K, Staugaitis SM, Steele R, Thorne L, Tirapelli DP, Unterberg A, Vallurupalli M, Wang Y, Warnick R, Williams F, Wolinsky Y, Bell S, Rosenberg M, Stewart C, Huang F, Grimsby JL, Radenbaugh AJ, Zhang J (2015). Comprehensive, integrative genomic analysis of diffuse lower-grade gliomas. New England Journal of Medicine.

[ref-4] Brennan CW, Verhaak RGW, McKenna A, Campos B, Noushmehr H, Salama SR, Zheng S, Chakravarty D, Sanborn JZ, Berman SH, Beroukhim R, Bernard B, Wu C-J, Genovese G, Shmulevich I, Barnholtz-Sloan J, Zou L, Vegesna R, Shukla SA, Ciriello G, Yung WK, Zhang W, Sougnez C, Mikkelsen T, Aldape K, Bigner DD, Van Meir EG, Prados M, Sloan A, Black KL, Eschbacher J, Finocchiaro G, Friedman W, Andrews DW, Guha A, Iacocca M, O’Neill BP, Foltz G, Myers J, Weisenberger DJ, Penny R, Kucherlapati R, Perou CM, Hayes DN, Gibbs R, Marra M, Mills GB, Lander E, Spellman P, Wilson R, Sander C, Weinstein J, Meyerson M, Gabriel S, Laird PW, Haussler D, Getz G, Chin L, Network TR (2013). The somatic genomic landscape of glioblastoma. Cell.

[ref-5] Cao Y, Becker C, Lundwall A, Christensson A, Gadaleanu V, Lilja H, Bjartell A (2003). Expression of protein C inhibitor (PCI) in benign and malignant prostatic tissues. Prostate.

[ref-6] Ceccarelli M, Barthel FP, Malta TM, Sabedot TS, Salama SR, Murray BA, Morozova O, Newton Y, Radenbaugh A, Pagnotta SM, Anjum S, Wang J, Manyam G, Zoppoli P, Ling S, Rao AA, Grifford M, Cherniack AD, Zhang H, Poisson L, Carlotti Jr CG, Tirapelli DP, Rao A, Mikkelsen T, Lau CC, Yung WK, Rabadan R, Huse J, Brat DJ, Lehman NL, Barnholtz-Sloan JS, Zheng S, Hess K, Rao G, Meyerson M, Beroukhim R, Cooper L, Akbani R, Wrensch M, Haussler D, Aldape KD, Laird PW, Gutmann DH, Noushmehr H, Iavarone A, Verhaak RG (2016). Molecular profiling reveals biologically discrete subsets and pathways of progression in diffuse glioma. Cell.

[ref-7] Chan AK, Mao Y, Ng HK (2016). TP53 and histone H3.3 mutations in triple-negative lower-grade gliomas. New England Journal of Medicine.

[ref-8] Conway K, Edmiston SN, May R, Kuan PF, Chu H, Bryant C, Tse CK, Swift-Scanlan T, Geradts J, Troester MA, Millikan RC (2014). DNA methylation profiling in the Carolina Breast Cancer Study defines cancer subclasses differing in clinicopathologic characteristics and survival. Breast Cancer Research.

[ref-9] Crocker M, Ashley S, Giddings I, Petrik V, Hardcastle A, Aherne W, Pearson A, Bell BA, Zacharoulis S, Papadopoulos MC (2011). Serum angiogenic profile of patients with glioblastoma identifies distinct tumor subtypes and shows that TIMP-1 is a prognostic factor. Neuro-Oncology.

[ref-10] Duan S, Cermak L, Pagan JK, Rossi M, Martinengo C, Di Celle PF, Chapuy B, Shipp M, Chiarle R, Pagano M (2012). FBXO11 targets BCL6 for degradation and is inactivated in diffuse large B-cell lymphomas. Nature.

[ref-11] Ellis L, Atadja PW, Johnstone RW (2009). Epigenetics in cancer: targeting chromatin modifications. Molecular Cancer Therapeutics.

[ref-12] Esteller M, Garcia-Foncillas J, Andion E, Goodman SN, Hidalgo OF, Vanaclocha V, Baylin SB, Herman JG (2000). Inactivation of the DNA-repair gene MGMT and the clinical response of gliomas to alkylating agents. New England Journal of Medicine.

[ref-13] Fortenberry Y (2015). The role of serpins in tumor cell migration. Biological Chemistry.

[ref-14] Frescas D, Pagano M (2008). Deregulated proteolysis by the F-box proteins SKP2 and beta-TrCP: tipping the scales of cancer. Nature Reviews Cancer.

[ref-15] Gao YF, Zhu T, Mao CX, Liu ZX, Wang ZB, Mao XY, Li L, Yin JY, Zhou HH, Liu ZQ (2016). PPIC, EMP3 and CHI3L1 are novel prognostic markers for high grade glioma. International Journal of Molecular Sciences.

[ref-16] Hagelgans A, Jandeck C, Friedemann M, Donchin A, Richter S, Menschikowski M (2017). Identification of CpG sites of SERPINA5 promoter with opposite methylation patterns in benign and malignant prostate cells. Anticancer Research.

[ref-17] Hegi ME, Diserens A, Gorlia T, Hamou M, De Tribolet N, Weller M, Kros JM, Hainfellner JA, Mason W, Mariani L, Bromberg JEC, Hau P, Mirimanoff RO, Cairncross JG, Janzer RC, Stupp R (2005). MGMT gene silencing and benefit from temozolomide in glioblastoma. New England Journal of Medicine.

[ref-18] Herman JG, Jen J, Merlo A, Baylin SB (1996). Hypermethylation-associated inactivation indicates a tumor suppressor role for p15INK4B. Cancer Research.

[ref-19] Huang da W, Sherman BT, Lempicki RA (2009). Systematic and integrative analysis of large gene lists using DAVID bioinformatics resources. Nature Protocols.

[ref-20] Jackson HW, Defamie V, Waterhouse P, Khokha R (2017). TIMPs: versatile extracellular regulators in cancer. Nature Reviews Cancer.

[ref-21] Jing Y, Jia D, Wong CM, Oi-Lin Ng I, Zhang Z, Liu L, Wang Q, Zhao F, Li J, Yao M, Wu X, He X (2014). SERPINA5 inhibits tumor cell migration by modulating the fibronectin-integrin beta1 signaling pathway in hepatocellular carcinoma. Molecular Oncology.

[ref-22] Lee SY, Kim JM, Cho SY, Kim HS, Shin HS, Jeon JY, Kausar R, Jeong SY, Lee YS, Lee MA (2014). TIMP-1 modulates chemotaxis of human neural stem cells through CD63 and integrin signalling. Biochemical Journal.

[ref-23] Louis DN, Ohgaki H, Wiestler OD, Cavenee WK, Burger PC, Jouvet A, Scheithauer BW, Kleihues P (2007). The 2007 WHO classification of tumours of the central nervous system. Acta Neuropathologica.

[ref-24] Louis DN, Perry A, Burger P, Ellison DW, Reifenberger G, Von Deimling A, Aldape K, Brat D, Collins VP, Eberhart C, Figarella-Branger D, Fuller GN, Giangaspero F, Giannini C, Hawkins C, Kleihues P, Korshunov A, Kros JM, BeatrizLopes M, Ng HK, Ohgaki H, Paulus W, Pietsch T, Rosenblum M, Rushing E, Soylemezoglu F, Wiestler O, Wesseling P (2014). International Society Of Neuropathology–Haarlem consensus guidelines for nervous system tumor classification and grading. Brain Pathology.

[ref-25] Louis DN, Perry A, Reifenberger G, von Deimling A, Figarella-Branger D, Cavenee WK, Ohgaki H, Wiestler OD, Kleihues P, Ellison DW (2016). The 2016 World Health Organization Classification of Tumors of the Central Nervous System: a summary. Acta Neuropathologica.

[ref-26] Lu C, Ward PS, Kapoor GS, Rohle D, Turcan S, Abdel-Wahab O, Edwards CR, Khanin R, Figueroa ME, Melnick A, Wellen KE, O’Rourke DM, Berger SL, Chan TA, Levine RL, Mellinghoff IK, Thompson CB (2012). IDH mutation impairs histone demethylation and results in a block to cell differentiation. Nature.

[ref-27] Luo WM, Wang ZY, Zhang X (2018). Identification of four differentially methylated genes as prognostic signatures for stage I lung adenocarcinoma. Cancer Cell International.

[ref-28] Merlo A, Herman JG, Mao L, Lee DJ, Gabrielson E, Burger PC, Baylin SB, Sidransky D (1995). 5’ CpG island methylation is associated with transcriptional silencing of the tumour suppressor p16/CDKN2/MTS1 in human cancers. Nature Medicine.

[ref-29] Noushmehr H, Weisenberger DJ, Diefes K, Phillips HS, Pujara K, Berman BP, Pan F, Pelloski CE, Sulman EP, Bhat KP, Verhaak RGW, Hoadley KA, Hayes DN, Perou CM, Schmidt HK, Ding L, Wilson RK, Van Den Berg D, Shen H, Bengtsson H, Neuvial P, Cope LM, Buckley J, Herman JG, Baylin SB, Laird PW, Aldape K, The Cancer Genome Atlas Research Network (2010). Identification of a CpG Island methylator phenotype that defines a distinct subgroup of glioma. Cancer Cell.

[ref-30] Ricard D, Idbaih A, Ducray F, Lahutte M, Hoang-Xuan K, Delattre JY (2012). Primary brain tumours in adults. Lancet.

[ref-31] Robinson MD, McCarthy DJ, Smyth GK (2010). edgeR: a Bioconductor package for differential expression analysis of digital gene expression data. Bioinformatics.

[ref-32] Sanson M, Marie Y, Paris S, Idbaih A, Laffaire J, Ducray F, El Hallani S, Boisselier B, Mokhtari K, Hoang-Xuan K, Delattre JY (2009). Isocitrate dehydrogenase 1 codon 132 mutation is an important prognostic biomarker in gliomas. Journal of Clinical Oncology.

[ref-33] Smith HW, Marshall CJ (2010). Regulation of cell signalling by uPAR. Nature Reviews Molecular Cell Biology.

[ref-34] Soini Y, Satta J, Maatta M, Autio-Harmainen H (2001). Expression of MMP2, MMP9, MT1-MMP, TIMP1, and TIMP2 mRNA in valvular lesions of the heart. Journal of Pathology.

[ref-35] Sreekanthreddy P, Srinivasan H, Kumar DM, Nijaguna MB, Sridevi S, Vrinda M, Arivazhagan A, Balasubramaniam A, Hegde AS, Chandramouli BA, Santosh V, Rao MR, Kondaiah P, Somasundaram K (2010). Identification of potential serum biomarkers of glioblastoma: serum osteopontin levels correlate with poor prognosis. Cancer Epidemiology, Biomarkers & Prevention.

[ref-36] Suzuki H, Aoki K, Chiba K, Sato Y, Shiozawa Y, Shiraishi Y, Shimamura T, Niida A, Motomura K, Ohka F, Yamamoto T, Tanahashi K, Ranjit M, Wakabayashi T, Yoshizato T, Kataoka K, Yoshida K, Nagata Y, Sato-Otsubo A, Tanaka H, Sanada M, Kondo Y, Nakamura H, Mizoguchi M, Abe T, Muragaki Y, Watanabe R, Ito I, Miyano S, Natsume A, Ogawa S (2015). Mutational landscape and clonal architecture in grade II and III gliomas. Nature Genetics.

[ref-37] Van den Bent MJ, Dubbink HJ, Marie Y, Brandes AA, Taphoorn MJ, Wesseling P, Frenay M, Tijssen CC, Lacombe D, Idbaih A, Van Marion R, Kros JM, Dinjens WN, Gorlia T, Sanson M (2010). IDH1 and IDH2 mutations are prognostic but not predictive for outcome in anaplastic oligodendroglial tumors: a report of the European Organization for Research and Treatment of Cancer Brain Tumor Group. Clinical Cancer Research.

[ref-38] Verhaak RGW, Hoadley KA, Purdom E, Wang V, Qi Y, Wilkerson MD, Miller CR, Ding L, Golub T, Mesirov JP, Alexe G, Lawrence M, O’Kelly M, Tamayo P, Weir BA, Gabriel S, Winckler W, Gupta S, Jakkula L, Feiler HS, Hodgson JG, James CD, Sarkaria JN, Brennan C, Kahn A, Spellman PT, Wilson RK, Speed TP, Gray JW, Meyerson M, Getz G, Perou CM, Hayes DN, The Cancer Genome Atlas Research Network (2010). Integrated genomic analysis identifies clinically relevant subtypes of glioblastoma characterized by abnormalities in PDGFRA, IDH1, EGFR, and NF1. Cancer Cell.

[ref-39] Wang X, Xu Z, Chen X, Ren X, Wei J, Zhou S, Yang X, Zeng S, Qian L, Wu G, Gong Z, Yan Y (2019). A tropomyosin receptor kinase family protein, NTRK2 is a potential predictive biomarker for lung adenocarcinoma. PeerJ.

[ref-40] Warde-Farley D, Donaldson SL, Comes O, Zuberi K, Badrawi R, Chao P, Franz M, Grouios C, Kazi F, Lopes CT, Maitland A, Mostafavi S, Montojo J, Shao Q, Wright G, Bader GD, Morris Q (2010). The GeneMANIA prediction server: biological network integration for gene prioritization and predicting gene function. Nucleic Acids Research.

[ref-41] Weller M, Felsberg J, Hartmann C, Berger H, Steinbach JP, Schramm J, Westphal M, Schackert G, Simon M, Tonn JC, Heese O, Krex D, Nikkhah G, Pietsch T, Wiestler O, Reifenberger G, von Deimling A, Loeffler M (2009). Molecular predictors of progression-free and overall survival in patients with newly diagnosed glioblastoma: a prospective translational study of the German Glioma Network. Journal of Clinical Oncology.

[ref-42] Weller M, Weber RG, Willscher E, Riehmer V, Hentschel B, Kreuz M, Felsberg J, Beyer U, Loeffler-Wirth H, Kaulich K, Steinbach JP, Hartmann C, Gramatzki D, Schramm J, Westphal M, Schackert G, Simon M, Martens T, Bostroem J, Hagel C, Sabel M, Krex D, Tonn JC, Wick W, Noell S, Schlegel U, Radlwimmer B, Pietsch T, Loeffler M, von Deimling A, Binder H, Reifenberger G (2015). Molecular classification of diffuse cerebral WHO grade II/III gliomas using genome- and transcriptome-wide profiling improves stratification of prognostically distinct patient groups. Acta Neuropathologica.

[ref-43] Wong JH, Dukes J, Levy RE, Sos B, Mason SE, Fong TS, Weiss EJ (2008). Sex differences in thrombosis in mice are mediated by sex-specific growth hormone secretion patterns. Journal of Clinical Investigation.

[ref-44] Xu Y, Geng R, Yuan F, Sun Q, Liu B, Chen Q (2019). Identification of differentially expressed key genes between glioblastoma and low-grade glioma by bioinformatics analysis. PeerJ.

[ref-45] Yan H, Parsons DW, Jin G, McLendon R, Rasheed BA, Yuan W, Kos I, Batinic-Haberle I, Jones S, Riggins GJ, Friedman H, Friedman A, Reardon D, Herndon J, Kinzler KW, Velculescu VE, Vogelstein B, Bigner DD (2009). IDH1 and IDH2 mutations in gliomas. New England Journal of Medicine.

[ref-46] Yan W, Zhang W, You G, Zhang J, Han L, Bao Z, Wang Y, Liu Y, Jiang C, Kang C, You Y, Jiang T (2012). Molecular classification of gliomas based on whole genome gene expression: a systematic report of 225 samples from the Chinese Glioma Cooperative Group. Neuro-Oncology.

[ref-47] Yan Y, Xu Z, Qian L, Zeng S, Zhou Y, Chen X, Wei J, Gong Z (2019). Identification of CAV1 and DCN as potential predictive biomarkers for lung adenocarcinoma. American Journal of Physiology–Lung Cellular and Molecular Physiology.

[ref-48] Yang H, Geiger M (2017). Cell penetrating SERPINA5 (ProteinC inhibitor, PCI): more questions than answers. Seminars in Cell and Developmental Biology.

[ref-49] Zeng WJ, Yang YL, Liu ZZ, Wen ZP, Chen YH, Hu XL, Cheng Q, Xiao J, Zhao J, Chen XP (2018). Integrative analysis of DNA methylation and gene expression identify a three-gene signature for predicting prognosis in lower-grade gliomas. Cellular Physiology and Biochemistry.

[ref-50] Zhang X, Lv QL, Huang YT, Zhang LH, Zhou HH (2017). Akt/FoxM1 signaling pathway-mediated upregulation of MYBL2 promotes progression of human glioma. Journal of Experimental & Clinical Cancer Research.

