# Supplementary figures and images for: Identification of gene expression and DNA methylation of SERPINA5 and TIMP1 as novel prognostic markers in lower-grade gliomas
Source: PeerJ. 2020 Jun 3;8:e9262. doi: 10.7717/peerj.9262 (PMC7275683; doi:10.7717/peerj.9262)

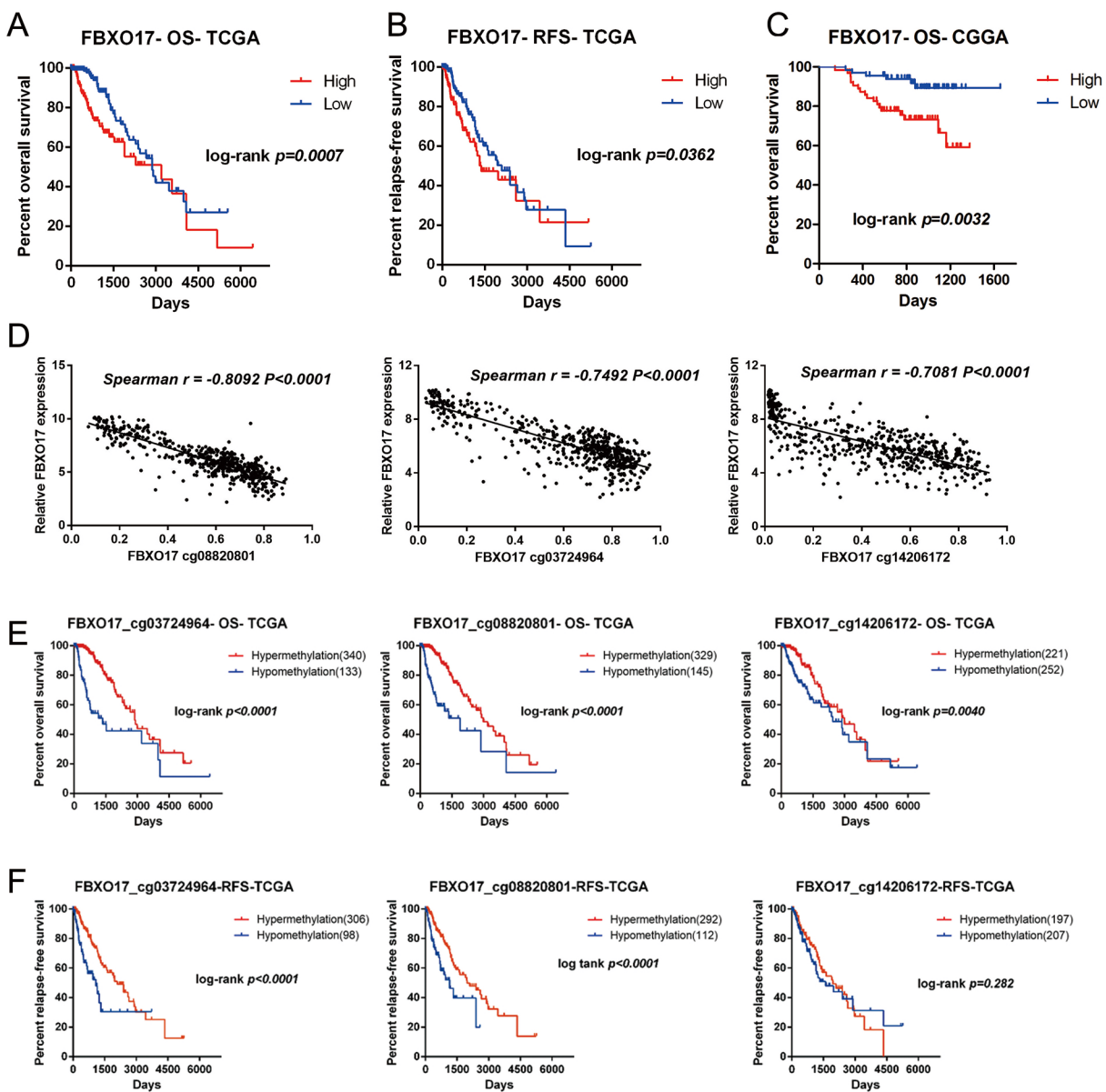

Supplement: Figure S1 — (A–C) Kaplan–Meier plot for survival between patients with high level and low level of FBXO17 expression in TCGA LGG and CGGA LGG dataset. (D) DNA methylation of FBXO17 CpG sites correlates with its gene expression in TCGA LGG dataset. (E, F) Methylation of FBXO17 CpG sites is associated with survival of LGGs patients in TCGA LGG dataset [file peerj-08-9262-s001.pdf]
