# Supplementary material for: Identification of gene expression and DNA methylation of SERPINA5 and TIMP1 as novel prognostic markers in lower-grade gliomas
Source: PeerJ. 2020 Jun 3;8:e9262. doi: 10.7717/peerj.9262 (PMC7275683; doi:10.7717/peerj.9262)
Supplement: Table S1 [file peerj-08-9262-s002.docx]

**Supplementary Table 1. Gene ontology analysis of DEGs in LGGs.**

| Category | Term | Count | PValue | Genes |
| --- | --- | --- | --- | --- |
| GOTERM_BP_DIRECT | GO:0007275~multicellular organism development | 16 | 1.22E-07 | WNT16, HMX1, DLL3, HMGA2, PAX1, HOXB3, EYA4, WNT7B, HOXA3, HILS1, MEOX2, HOXA4, HOXA5, SFRP2, CUX2, TLX1 |
| GOTERM_BP_DIRECT | GO:0030878~thyroid gland development | 4 | 3.25E-04 | HOXB3, HOXA3, HOXA5, NKX2-5 |
| GOTERM_BP_DIRECT | GO:0009952~anterior/posterior pattern specification | 5 | 9.32E-04 | HOXB3, HOXA2, HOXA3, HOXA5, HOXA7 |
| GOTERM_BP_DIRECT | GO:0048704~embryonic skeletal system morphogenesis | 4 | 0.001222 | HOXB3, HOXA3, HOXA5, HOXA7 |
| GOTERM_BP_DIRECT | GO:0051216~cartilage development | 4 | 0.00402 | HOXB3, HOXA3, CHI3L1, TIMP1 |
| GOTERM_BP_DIRECT | GO:0045669~positive regulation of osteoblast differentiation | 4 | 0.004215 | WNT7B, SFRP2, NPNT, LTF |
| GOTERM_BP_DIRECT | GO:0001525~angiogenesis | 6 | 0.007289 | HOXB3, VAV3, HOXA3, MEOX2, HOXA7, TMEM100 |
| GOTERM_BP_DIRECT | GO:0050482~arachidonic acid secretion | 3 | 0.007419 | ANXA1, PLA2G2A, PLA2G5 |
| GOTERM_BP_DIRECT | GO:0060349~bone morphogenesis | 3 | 0.009336 | SFRP2, LTF, PAX1 |
| GOTERM_BP_DIRECT | GO:0072272~proximal/distal pattern formation involved in metanephric nephron development | 2 | 0.010809 | IRX2, IRX1 |
| GOTERM_BP_DIRECT | GO:0016055~Wnt signaling pathway | 5 | 0.018791 | WNT16, WNT7B, WISP1, SFRP2, LGR6 |
| GOTERM_BP_DIRECT | GO:0021615~glossopharyngeal nerve morphogenesis | 2 | 0.021503 | HOXB3, HOXA3 |
| GOTERM_BP_DIRECT | GO:0072086~specification of loop of Henle identity | 2 | 0.021503 | IRX2, IRX1 |
| GOTERM_BP_DIRECT | GO:0060535~trachea cartilage morphogenesis | 2 | 0.021503 | WNT7B, HOXA5 |
| GOTERM_BP_DIRECT | GO:0061056~sclerotome development | 2 | 0.021503 | SFRP2, PAX1 |
| GOTERM_BP_DIRECT | GO:0030198~extracellular matrix organization | 5 | 0.0219 | HPSE2, ADAMTS20, NPNT, POSTN, LOXL1 |
| GOTERM_BP_DIRECT | GO:0007165~signal transduction | 13 | 0.02257 | KLRC2, PDPN, ANXA1, CNGA3, HMGA2, WISP1, STAC, TOM1L1, CXCL14, SMOC1, IGFBP2, GDF15, CLEC5A |
| GOTERM_BP_DIRECT | GO:0045165~cell fate commitment | 3 | 0.025762 | WNT16, WNT7B, NEUROD4 |
| GOTERM_BP_DIRECT | GO:0007187~G-protein coupled receptor signaling pathway, coupled to cyclic nucleotide second messenger | 3 | 0.025762 | ANXA1, GPR1, TSHR |
| GOTERM_BP_DIRECT | GO:0002138~retinoic acid biosynthetic process | 2 | 0.026807 | RBP1, ALDH1A3 |
| GOTERM_BP_DIRECT | GO:0006355~regulation of transcription, DNA-templated | 15 | 0.031353 | IRX2, IRX1, DMRTA2, HMGA2, OTP, HOXD11, HOXB3, SHOX2, EYA4, HOXA3, HILS1, HOXA4, MEOX2, NEUROD4, TLX1 |
| GOTERM_BP_DIRECT | GO:0043066~negative regulation of apoptotic process | 7 | 0.037107 | ADAMTS20, ANXA1, LTF, HMGA2, CLEC5A, NKX2-5, TIMP1 |
| GOTERM_BP_DIRECT | GO:0060017~parathyroid gland development | 2 | 0.03733 | HOXA3, PAX1 |
| GOTERM_BP_DIRECT | GO:0060428~lung epithelium development | 2 | 0.03733 | WNT7B, HMGA2 |
| GOTERM_BP_DIRECT | GO:0001501~skeletal system development | 4 | 0.038384 | SHOX2, DLL3, POSTN, PAX1 |
| GOTERM_BP_DIRECT | GO:0009612~response to mechanical stimulus | 3 | 0.040712 | CHI3L1, POSTN, IGFBP2 |
| GOTERM_BP_DIRECT | GO:0007267~cell-cell signaling | 5 | 0.049269 | WISP1, CXCL14, SFRP2, GDF15, TSHR |
| GOTERM_CC_DIRECT | GO:0005578~proteinaceous extracellular matrix | 14 | 1.53E-09 | FMOD, WNT16, CRTAC1, ADAMTS20, NPNT, COL22A1, CHI3L1, POSTN, TIMP1, WNT7B, WISP1, HPSE2, SMOC1, FBLN7 |
| GOTERM_CC_DIRECT | GO:0005615~extracellular space | 20 | 6.58E-05 | VASN, FMOD, WNT16, ADAMTS20, ANXA1, CHI3L1, POSTN, TIMP1, WNT7B, WISP1, CXCL14, SFRP2, SAA1, SERPINA5, PLA2G2A, LTF, IGFBP2, GDF15, LOXL1, IL13RA2 |
| GOTERM_CC_DIRECT | GO:0005576~extracellular region | 21 | 2.33E-04 | FMOD, RARRES2, WNT16, NPNT, COL22A1, ANXA1, TIMP1, WNT7B, CXCL14, SFRP2, SAA1, C5ORF38, SERPINA5, PLA2G2A, LTF, IGFBP2, ADAM12, GDF15, LOXL1, PLA2G5, IL13RA2 |
| GOTERM_CC_DIRECT | GO:0070062~extracellular exosome | 27 | 0.00232 | GALNT3, RARRES2, NPNT, AQP5, TIMP1, SPHKAP, DES, SAA1, ALDH1A3, SERPINA5, LTF, VASN, VAV3, CRTAC1, MAOB, ANXA1, CHI3L1, WNT7B, DSG2, TOM1L1, FBLN7, PLA2G2A, GFRA1, CUX2, IGFBP2, GDF15, FABP5 |
| GOTERM_CC_DIRECT | GO:0005604~basement membrane | 4 | 0.00839 | SMOC1, NPNT, LOXL1, TIMP1 |
| GOTERM_CC_DIRECT | GO:0031012~extracellular matrix | 6 | 0.020153 | FMOD, RARRES2, ADAMTS20, SFRP2, POSTN, LOXL1 |
| GOTERM_CC_DIRECT | GO:0048471~perinuclear region of cytoplasm | 8 | 0.045595 | GALNT3, RYR3, CMYA5, PLA2G2A, TSTD1, CHI3L1, TMEM100, PLA2G5 |
| GOTERM_MF_DIRECT | GO:0043565~sequence-specific DNA binding | 15 | 7.72E-07 | IRX2, IRX1, DMRTA2, HOXD11, OTP, SHOX2, HOXB3, HOXA3, HOXA4, MEOX2, HOXA5, HOXA7, CUX2, NKX2-5, TLX1 |
| GOTERM_MF_DIRECT | GO:0008201~heparin binding | 8 | 2.69E-05 | FMOD, WISP1, SAA1, SERPINA5, FBLN7, LTF, POSTN, PLA2G5 |
| GOTERM_MF_DIRECT | GO:0005509~calcium ion binding | 11 | 0.005608 | GALNT3, DSG2, CRTAC1, SMOC1, RYR3, NPNT, ANXA1, PLA2G2A, FBLN7, DLL3, PLA2G5 |
| GOTERM_MF_DIRECT | GO:0001077~transcriptional activator activity, RNA polymerase II core promoter proximal region sequence-specific binding | 6 | 0.009404 | MEOX2, HOXA5, HOXA7, HMGA2, NKX2-5, TLX1 |
| GOTERM_MF_DIRECT | GO:0000978~RNA polymerase II core promoter proximal region sequence-specific DNA binding | 7 | 0.013016 | HOXA2, MEOX2, HOXA5, HOXA7, CUX2, NKX2-5, TLX1 |
| GOTERM_MF_DIRECT | GO:0047498~calcium-dependent phospholipase A2 activity | 2 | 0.048005 | PLA2G2A, PLA2G5 |
