# Supplementary material for: Identification of gene expression and DNA methylation of SERPINA5 and TIMP1 as novel prognostic markers in lower-grade gliomas
Source: PeerJ. 2020 Jun 3;8:e9262. doi: 10.7717/peerj.9262 (PMC7275683; doi:10.7717/peerj.9262)
Supplement: Table S2 [file peerj-08-9262-s003.docx]

**Supplementary Table 2. Characteristics of the selected DNA methylation probes**

| **Gene symbol** | **CG sites** | **Chr** | **MAPINFO** | **Location** |
| --- | --- | --- | --- | --- |
| SERPINA5 | cg15509705 | 14 | 95047720 | 1stExon;5URT |
| TIMP1 | cg27151711 | X | 47441573 | TSS200;Body;Body |
| TIMP1 | cg16523424 | X | 47441779 | 1stExon;5URT;Body;Body |
| TIMP1 | cg04791822 | X | 47441773 | 1stExon;5URT;Body;Body |
